# Endothelial Progenitor Cells and Cardiovascular Events in Patients with Chronic Kidney Disease – a Prospective Follow-Up Study

**DOI:** 10.1371/journal.pone.0011477

**Published:** 2010-07-08

**Authors:** Johan Lorenzen, Sascha David, Ferdinand H. Bahlmann, Kirsten de Groot, Elisabeth Bahlmann, Jan T. Kielstein, Hermann Haller, Danilo Fliser

**Affiliations:** 1 Division of Nephrology, Department of Internal Medicine, Hannover Medical School, Hannover, Germany; 2 Department of Internal Medicine IV, Saarland University Medical Centre, Homburg/Saar, Germany; L' Istituto di Biomedicina ed Immunologia Molecolare, Consiglio Nazionale delle Ricerche, Italy

## Abstract

**Background:**

Endothelial progenitor cells (EPCs) mediate vascular repair and regeneration. Their number in peripheral blood is related to cardiovascular events in individuals with normal renal function.

**Methods:**

We evaluated the association between functionally active EPCs (cell culture) and traditional cardiovascular risk factors in 265 patients with chronic kidney disease stage V receiving hemodialysis therapy. Thereafter, we prospectively assessed cardiovascular events, e.g. myocardial infarction, percutaneous transluminal coronary angioplasty (including stenting), aorto-coronary bypass, stroke and angiographically verified stenosis of peripheral arteries, and cardiovascular death in this cohort.

**Results:**

In our patients EPCs were related only to age (r = 0.154; p = 0.01). During a median follow-up period of 36 months 109 (41%) patients experienced a cardiovascular event. In a multiple Cox regression analysis, we identified EPCs (p = 0.03) and patient age (p = 0.01) as the only independent variables associated with incident cardiovascular events. Moreover, a total of 70 patients died during follow-up, 45 of those due to cardiovascular causes. Log rank test confirmed statistical significance for EPCs concerning incident cardiovascular events (p = 0.02).

**Conclusions:**

We found a significant association between the number of functionally active EPCs and cardiovascular events in patients with chronic kidney disease. Thus, defective vascular repair and regeneration may be responsible, at least in part, for the enormous cardiovascular morbidity in this population.

## Introduction

Endothelial progenitor cells (EPCs) mediate reparative processes in the cardiovascular (CV) system [1], [2]. They originate, at least in part, from bone-marrow related CD34+ hematopoetic stem cells and circulate in the vasculature where they home and incorporate into sites of active neo-vascularization. The existence of EPC niches outside the bone-marrow has been proposed [3]. In the general population as well as in patients with coronary artery disease the number of EPCs correlates significantly with traditional CV risk factors such as blood pressure [4], [5]. Moreover, Hill et al. [4] could demonstrate that the number of EPCs was a better predictor of the non-invasively assessed endothelial function in healthy men than the combined Framingham risk score. Above that, prospective studies revealed that reduced EPC numbers are a significant independent predictor of future CV events (CVE) and of poor prognosis, even after adjustment for traditional CV risk factors [6], [7].

We and others have shown that the number and function of EPCs is reduced in uremic patients with advanced kidney failure before treatment of renal anemia with erythropoietin [8]–[12]. Attenuation of uremic intoxication by hemodialysis (HD) or kidney transplantation increases EPC numbers and improves their function [8], [11], [12]. Since patients with chronic kidney disease (CKD) represent a high risk population with regard to CVE [13], [14], we set out to explore if EPC numbers correlate with traditional CV risk factors in a cohort of 265 stable CKD stage V patients on maintenance HD. Moreover, we studied the association between EPCs and incident CVE as well as survival during a prospective follow-up.

## Results

We completed a total of 265 data sets from CKD patients on maintenance HD. Patient characteristics are shown in **Table 1**. In this baseline cohort we found a significant correlation between EPCs and age (r = 0.154; p = 0.01), but not with other traditional CV risk factors such as hsCRP, total serum cholesterol or blood pressure. A total of 231 patients received rHuEPO (87%) and 150 patients were on statins (57%). Patients on rHuEPO treatment had comparable EPCs as those without (419±18 vs. 461±42 per high power field; p = 0.4). Similarly, we found no significant difference in EPCs in CKD patients on statins compared to those without statin therapy (421±21 vs. 433±27 per high power field; p = 0.7). There was no association between rHuEPO (p = 0.287) and statin (p = 0.594) treatment and EPCs.

**Table 1:** Clinical and laboratory data of renal patients with and without incident cardiovascular events (CVE) during follow-up.

	All patients	Incident CVE	No CVE	p-value
Number	265	109	156	
Age (years)	66 (15)	70 (12)	66 (19)	0.02*
Male / female	147/118	64/45	83/73	0.37
Body mass index (kg/m^2^)	25.2 (5.8)	25.2 (5.9)	25.5 (5.6)	0.75
Dialysis vintage prior inclusion (months)	39 (68)	38 (51)	39 (73)	0.98
Diabetes (n)	91	40	51	0.5
Peripheral artery disease (n)	95	48	47	0.02*
Coronary artery disease (n)	129	65	64	0.02*
Hypertension (n)	217	90	127	0.69
Patients on statins (n)	150	70	80	0.07
Patients on AT_1_-antagonists	59	26	33	0.56
Patients on ACE-inhibitors	77	45	32	0.92
Patients on Beta-blockers	86	43	43	0.12
Patients on Calcium-channel-blockers	45	25	20	0.49
Patients on EPO	231	95	136	0.85
EPO-dose (IU/week)	6000 (6000)	6000 (4000)	6000 (5000)	0.18
Current smoker (n)	26	12	14	0.58
MAP (mmHg)	97 (14)	97 (18)	97 (13)	0.94
High sensitivity C-reactive protein (mg/L)	3.9 (6.9)	3.2 (6.2)	4.3 (7)	0.80
Serum total cholesterol (mg/dL)	172 (2.6)	176 (4.3)	168 (3.2)	0.22
Serum triglycerides (mg/dL)	169 (132)	188 (166)	158 (116)	0.56
Serum albumin (g/L)	40 (5)	40 (6)	39 (5)	0.83
EPCs(per high power field)	360 (338)	329 (256)	397 (403)	0.02*
HSCs (per μl)	1.4 (1.11)	1.5 (0.96)	1.4 (1.34)	0.39

MAP  =  mean arterial blood pressure; EPCs  =  endothelial progenitor cells; HSCs  =  CD34+ hematopoetic stem cells. Except for serum total cholesterol levels data are displayed as median (interquartile range); *p<0.05

**Figure 1:**
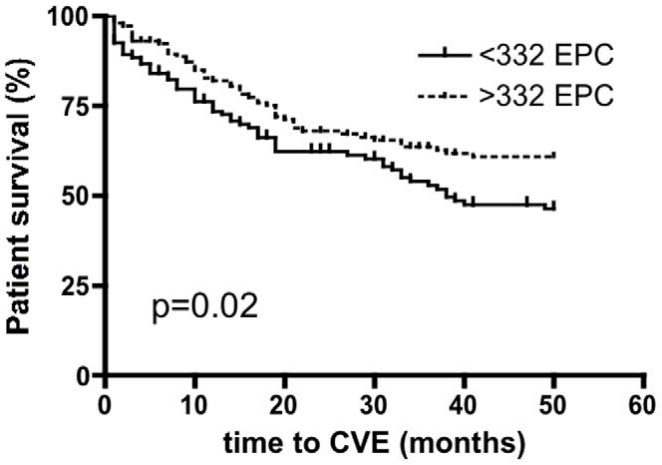
Kaplan-Meier curves for incident CVE in 265 stable patients with chronic kidney disease stage V receiving maintenance hemodialysis. Patients were compared above and below a cut-point of EPC numbers identified by ROC-curve analysis. In the follow-up period of 36 [1–54] months 109 patients experienced a CVE. Log rank testing confirmed statistical significance for EPCs with respect to incident CVE (p = 0.02).

During the median follow-up period of 36 [1–54] months 109 (41%) patients experienced a CVE. Forty-five patients experienced a myocardial infarction (17%), 13 patients a stroke (5%), 48 patients underwent a percutaneous transluminal coronary angiography (18%), 5 patients a coronary bypass surgery (2%) and 51 patients showed an angiographically verified stenosis of peripheral arteries (19%). In addition, a total of 70 patients died during the follow-up period, 45 of those due to a confirmed CV cause. In that group 25 patients died due to a myocardial infarction (9%), 10 patients due to a stroke (4%), 10 in the course of a bypass surgery (4%). Moreover, 17 patients died due to sepsis (6%), 1 due to Creutzfeld-Jacob disease (0.4%), 1 due to a metastasized protaste cancer (0.4%), 1 due to a car accident (0.4%), 2 due to pneumonia (0.8%), 3 in the nursing home (cause of death not known) (1.1%). In a multiple Cox-regression analysis performed with CV risk factors listed in **Table 2**, we identified EPCs (p = 0.03) and age (p = 0.01) as the only independent variables associated with incident CVE, whereas HSCs were not associated with incident CVE. We performed a ROC-curve analysis (AUC 0.6, 96% CI 0.5–0.64, p = 0.03) to determine a cut-off value, which separates patients with and without incident CVE with respect to EPCs. Clinical characteristics of patients above and below the cut-off value are shown in **Table 3**. A cut-off value of 332 cells per high power field separated patients with and without incident CVE with a sensitivity of 61% and a specificity of 53%. Using log rank testing we could confirm statistical significance for EPCs with respect to incident CVE stratified to values above and below this cut-point (p = 0.02) (**Figure 1**). In contrast, EPCs were not predictive for patient survival (p = 0.12). HSCs were not predictive for incident CVE (p = 0.6) and patient survival (p = 0.4).

**Table 2:** Simple and multiple Cox-Regression analysis concerning incident cardiovascular events (CVE) during follow-up.

		Univariate			Multivariate	
	HR	95% CI	p-value	HR	95% CI	p-value
Age (years)	1.018	1.003 to 1.034	0.02*	1.021	1.005 to 1.037	0.01*
EPCs (per high power field)	0.999	0.998 to 1.000	0.04*	0.999	0.998 to 1.000	0.03*
Serum Cholesterol (mg/dL)	1.005	1.000 to 1.009	0.05*	1.004	0.999 to 1.008	0.12
Sex (male/female)	1.165	0.795 to 1.705	0.43			
Body mass index (kg/m^2^)	1.002	0.965 to 1.041	0.91			
Dialysis vintage (months)	1	0.998 to 1.003	0.8			
Diabetes	1.179	0.799 to 1.740	0.41			
Current smoker	0.975	0.523 to 1.819	0.94			
MAP (mmHg)	0.999	0.988 to 1.011	0.93			
hsC-RP (mg/L)	1.005	0.992 to 1.018	0.45			
Triglycerides (mg/dL)	1.001	1.000 to 1.002	0.22			
Serum albumin (g/L)	0.992	0.949 to 1.038	0.73			
HSCs (per μl)	0.939	0.803 to 1.099	0.43			

EPCs  =  endothelial progenitor cells;

HSCs  =  CD34+ hematopoetic stem cells;

MAP  =  mean arterial blood pressure;

hsC-RP  =  high-sensitive C-reactive protein;

HR  =  hazard ratio;

CI  =  confidence interval. *p<0.05

**Table 3:** Clinical and laboratory data of renal patients with low vs. high levels of endothelial progenitor cells (EPCs) (above and below 332 EPCs per high power field).

	EPC <332	EPC <332	p-value
Age (years)	66 (19)	67 (14)	0.29
Male / female	68/53	79/65	0.83
Body mass index (kg/m^2^)	25.7 (6)	25.2 (5.3)	0.65
Dialysis vintage prior inclusion (months)	44 (78)	34 (46)	0.05*
Diabetes (n)	36	55	0.15
Peripheral artery disease (n)	51	44	0.04*
Coronary artery disease (n)	56	73	0.51
Hypertension (n)	102	115	0.28
Patients on statins (n)	78	72	0.04*
Patients on AT_1_-antagonists	32	27	0.12
Patients on ACE-inhibitors	33	44	0.07
Patients on Beta-blockers	47	39	0.33
Patients on Calcium-channel-blockers	26	19	0.29
Patients on EPO	106	125	0.71
EPO-dose (IU/week)	6000 (5000)	7000 (5500)	0.01*
Current smoker (n)	6	20	0.02*
MAP (mmHg)	93 (18)	97 (14)	0.47
High sensitivity C-reactive protein (mg/L)	3.3 (6.2)	3.8 (7.1)	0.69
Serum total cholesterol (mg/dL)	176 (3.9)	168 (3.5)	0.10
Serum triglycerides (mg/dL)	186 (152)	164 (124)	0.09
Serum albumin (g/L)	40 (4)	40 (5)	0.26
EPCs(per high power field)	238 (130)	514 (379)	<0.0001*
HSCs (per μl)	1.4 (1.1)	1.4 (1.0)	0.96
Time to first CVE (months)	33 (40)	37 (36)	0.10

HSCs  =  CD34+ hematopoetic stem cells. Except for serum total cholesterol levels data are displayed as median (interquartile range);

* p<0.05

## Discussion

In the present study we found a significant relationship between the number of EPCs and incident CVE in 265 stable patients with CKD stage V on maintenance HD in a prospective follow-up period of a median of 36 months. Moreover, in a stepwise logistic regression analysis including traditional CV risk factors EPCs were the only independent variable associated with incident CVE. In contrast, HSCs (assessed by flow cytometry) were not associated with CVE and patient survival in the follow-up, highlighting the potential role of EPCs, i.e. cells that are committed to the endothelial lineage, in vascular repair and CV morbidity in CKD patients. To our knowledge, this is the first study to show such a relationship in CKD patients after reports in CV high risk patients with normal kidney function [6], [7]. However, in contrast to individuals with normal kidney function we could find a significant but only weak correlation between EPCs and age, but not with other traditional CV risks factors such as high blood pressure or total serum cholesterol, and with CV risk factors present only in HD patients such as reduced nutritional status, e.g. low body mass index and/or serum albumin, and presence of (micro)inflammation, e.g. increased hsCRP. The latter finding is of particular important, since it supports the notion that EPCs obtained by labor intensive and time consuming cell culture methodology, thus exhibiting several functional properties of endothelial cells, are indeed related to vascular biology, and are not monocytic cells that may also be involved in inflammation.

Due to their endothelial and hence vascular repair potential EPCs have come into focus of CV research in the last decade. This has been shown in several experimental studies using different animal models of CV injury [1]–[3]. In humans, it has been shown that the number of circulating EPCs correlates with non-invasively assessed endothelial function [4], and with the number of CV risk factors in individuals with and without manifest CV disease [4], [5]. Hill et al. [4] found a significant negative correlation between EPC number and the Framingham risk factor score, i.e. a crude estimate of the risk of coronary artery disease, in asymptomatic men with established CV risk factors. It has been therefore hypothesized that traditional CV risk factors such as high blood pressure and serum cholesterol may impair EPC number and consequently their regenerative potential [15]. Moreover, a low CD34+ hematopoetic cell count was associated with CVE and CV death, independently of all potential confounders, in patients with metabolic syndrome [16]. Since CKD patients are characterized by high CV morbidity and mortality due to vascular complications, their survival could also be influenced, at least in part, by deficient vascular repair as a result of reduced EPC number and/or function [8], [11], [17]. This idea is supported by data of the present study.

Similar to us Steiner et al. [18] did not find an association between rHuEPO treatment and EPCs. This observation is of interest, since we and others could convincingly demonstrate that administration of rHuEPO is a strong stimulus for EPC mobilization and proliferation in-vitro and in-vivo [9], [19], [20]. However, the dose necessary for significant stimulation of EPC proliferation is much lower than that used for correction of anemia in CKD patients [9]. Thus, one could speculate that patients who do not need rHuEPO therapy despite kidney failure, still may have enough endogenous EPO production to maintain normal EPC numbers. In line with this speculation is the observation that patients after successful kidney transplantation have apparently normal EPC numbers and function [12], [21]. Other drugs beside rHuEPO are known to influence proliferation and differentiation of EPCs as well, e.g. statins [22] and angiotensin receptor blockers [23]. Again, we could not find differences in EPCs between patients treated with statins and those who did not receive these drugs. We therefore assume that other (unknown?) factors override the effect of rHuEPO and statins on EPCs in this population.

As a matter of fact, an important confounding factor may be the dialysis procedure by itself [8], [11]. Recent experimental studies revealed that EPCs represent a heterogeneous cell population of multiple origins and distinct phenotypes, but share the ability to differentiate into mature functionally competent endothelial cells [2], [3]. The prevailing view is that a small fraction of bone marrow derived CD34+ hematopoetic cells are the major source of EPCs, but EPCs can be also cultured from a CD14+ monocyte/macrophage population capable of secreting different cytokines and growth factors [3], [24], [25]. Thus, the dialysis procedure - particularly the blood-membrane contact - in combination with the (micro)inflammatory condition present in many CKD patients receiving HD may influence the biology (and number?) of this cell population, similarly as we and others have shown for mature monocytes [26]. Thus, we have studied EPCs in our patients after the long HD interval in order to minimize this potential confounding factor. As another limitation to our study we did not use the current state-of-the-art detection of EPCs including CD133, since this flow cytometry surface marker was not part of the standard panel for detection of EPCs when the study was initiated.

In conclusion, we found a significant relationship between EPCs and incident CVE as well as patient survival in stable CKD patients receiving HD therapy. Thus, our data support a role for EPCs in vascular repair in CKD patients. Future studies including patients at earlier stages of CKD are warranted to further elucidate the role of EPCs in chronic kidney disease.

## Methods

### Participants and protocol

The study protocol was approved by the Hannover Medical School Ethics Committee, and we conducted the study in adherence to the *Declaration of Helsinki*. We studied stable Caucasian patients with terminal renal failure from 6 HD centers in the region of Hannover, Germany. In order to avoid inter-observer bias all patients data were checked by two independent subjects who visited all participating centers. All patients gave their written informed consent for participation.

The study subjects were enrolled between October 2004 and January 2006. All patients studied were clinically stable and on maintenance HD for at least 3 months. Patients with concomitant acute inflammatory diseases, i.e. a high sensitivity C-reactive protein (hsCRP) above 20 mg/L, or clinically manifest acute infections, malignant diseases, manifest or occult bleeding conditions or recent CVE within 3 months prior to enrollment were excluded from the study. Patient history including dialysis vintage, co-morbidities, smoking habits, treatment with statins or recombinant human erythropoietin (rHuEPO) were recorded and confirmed by checking patients' records. The medical history was complemented by a physical examination including assessment of body mass index (BMI) and resting blood pressure prior to the start of the HD session. Arterial hypertension was defined as blood pressure above 140/90 mmHg or antihypertensive treatment.

EDTA blood samples for blood count, routine chemistry, EPC culture and flow cytometry (FACS-analysis) were drawn from the dialysis cannulas immediately before the HD session was started. We carried out these investigations in all patients after a two day dialysis-free interval (long interval). Part of the blood samples was used immediately for EPC culture and FACS-analysis; the rest was centrifuged at 1500× g at 4°C for 10 minutes and then stored at −80°C for further analysis. All routine laboratory measurements including hsCRP were done using certified assay methods.

After the initial screening, all patients entered a prospective follow-up (median of 36 [1–54] months). In this period we assessed all CVE defined as myocardial infarction, percutaneous transluminal coronary angioplasty (including stenting), aorto-coronary bypass, stroke and angiographically verified stenosis of peripheral arterial vessels (carotic, aorto-iliac or femoral arteries). Furthermore, we also assessed death to any cause including CV death.

### Isolation of EPCs

We isolated peripheral blood mononuclear cells from 14 ml blood in order to cultivate EPCs described in detail previously [8], [9]. In brief, we used density gradient centrifugation with Bicoll (Biochrome), and seeded 107 cells on 6-well plates coated with human fibronectin (Sigma) in endothelial basal medium (EBM-2, Clonetics). The medium was supplemented with EGM-2 Single Quots containing fetal bovine serum, human VEGF-A, human fibroblast growth factor-B, human epidermal growth factor, insulin-like growth factor-1 and ascorbic acid in appropriate amounts. After 4 days in culture, we removed non-adherent cells by washing the plates with PBS. We trypsinated the remaining adherent cells and reseeded 106 cells on fibronectin coated 6-well plates. New media was applied and the cell culture was maintained through day 7. We further performed fluorescent chemical detection in order to determine the cell type of the attached human peripheral blood mononuclear cells after 7 days in culture. To detect the uptake of 1,1′-dioctadecyl-3,3,3′,3′-tetramethylindocarbocyanine-labeled acetylated low density lipoprotein (acLDL-DiI, Molecular Probes), we incubated the cells with acLDL-DiI (6 µg/ml) at 37°C for 2 hours. Cells were then fixed with 1% para-formaldehyde for 10 minutes and incubated with FITC-labeled Ulex europaeus agglutinin-1 (UEA-1, Sigma) for 1 hour. After the staining, we viewed the samples with an inverted fluorescent microscope (Leica). We counted double stained cells for both UEA-1 and acLDL-DiI as EPCs. Two blinded investigators counted at least four randomly selected high-power fields.

### Flow cytometry of circulating hematopoetic stem cells

In all patients we analyzed the total number of circulating CD34+ hematopoetic stem cells (HSCs) using flow cytometry (*Epics XL cytometer*, Coulter Beckman). We adopted a gating strategy for flow cytometry on the basis of the ISHAGE guidelines, and used the CD34 and CD45 expression patterns as well as the morphological qualities of progenitor cells for their detection, as described in detail previously [9]. In brief, we stained whole EDTA blood within 6 hours after drawing the blood. Thereafter we incubated a volume of 100 µl with an appropriate amount of FITC-labeled monoclonal mouse anti-human-CD45 antibody (Coulter Beckman) for 20 minutes. For detection of HSCs we added PE-labeled monoclonal mouse anti-human-CD34 antibody (Coulter Beckman) to the sample after titration of the optimal antibody concentration. In addition, we added a PE-labeled mouse IgG1-antibody (Coulter Beckman) to a second anti-CD45 stained blood sample as the isotype control. Subsequent lysis was done with ammonium chloride, and at least 200.000 CD45+ cells were acquired. Two blinded investigators independently assessed the number of HSCs. Day-to-day variability of the absolute HSC number was below 12% as assessed by flow cytometry of HSCs in 8 subjects on 4 consecutive days. Inter-assay variability (n = 10) was below 5%.

### Statistical analysis

Statistical analysis was performed with Statistical Package for the Social Sciences (SPSS) 15.0 for Windows and GraphPad Prism software (GraphPad Prism Software Inc. San Diego, California, USA). Two-sided p-values <0.05 were considered statistically significant for all statistical procedures. Normally distributed continuous variables are expressed as mean ± SEM. All variables used for parametric statistical analyses were analyzed for normal distribution using Kolmogorov-Smirnov test. In case of non-normal distribution parameters are displayed as median [interquartile range]. Follow-up time is displayed as median [min–max]. Univariate comparisons of continuous variables between groups were performed using an unpaired t test or the nonparametric Wilcoxon rank sum test in case of non-normally distributed variables. Dichotomized variables were compared using Pearson's χ2-test. Univariate correlation analysis was performed by Spearman correlation analysis. In addition, parameters independently associated with incident CVE were identified by univariate and multivariate Cox proportional hazards models. Variables found to be statistically significant at a 10% level in the univariate analysis were included in the multivariate model using backward elimination. The distribution of the time-to-event variables were estimated using the Kaplan-Meier method with log-rank testing. Receiver operator characteristics (ROC) procedures were used to identify optimal cut-off values.
